# Microglia activation in central nervous system disorders: A review of recent mechanistic investigations and development efforts

**DOI:** 10.3389/fneur.2023.1103416

**Published:** 2023-03-07

**Authors:** Jun Qin, Zhihui Ma, Xiaoli Chen, Shiyu Shu

**Affiliations:** Department of Anesthesiology, The Second Affiliated Hospital of Chongqing Medical University, Chongqing, China

**Keywords:** microglia, activation, membrane receptor, signaling pathway, inflammatory response

## Abstract

Microglia are the principal resident immune cells in the central nervous system (CNS) and play important roles in the development of CNS disorders. In recent years, there have been significant developments in our understanding of microglia, and we now have greater insight into the temporal and spatial patterns of microglia activation in a variety of CNS disorders, as well as the interactions between microglia and neurons. A variety of signaling pathways have been implicated. However, to date, all published clinical trials have failed to demonstrate efficacy over placebo. This review summarizes the results of recent important studies and attempts to provide a mechanistic view of microglia activation, inflammation, tissue repair, and CNS disorders.

## Introduction

In 1856, the German pathologist RUDOLF (1) coined the term “glia” to describe a distinct population of cells, other than neurons, in the brain. In the following decades, research in this field progressed slowly. It was not until 1919 that microglia were accepted as an independent cell type (2). Microglia account for 5–20% of all glial cells in the central nervous system (CNS) and are resident immune cells in the CNS (3). Under normal physiological conditions, microglia are in a resting state, with a branched cellular arrangement; they monitor their surroundings and play an important role in the regulation and monitoring of neurological homeostasis, proliferation and regeneration of neurons, and nourishment of nerves (4). Upon infection or injury, microglia are activated and move toward the site of infection/injury, releasing a variety of cytotoxic substances to attack the infecting agents and clear the damaged cells. When microglia are activated abnormally, the released cytotoxic substances can damage the surrounding normal tissues (5). Inhibition of aberrant microglial activation could limit the degree of neuroinflammation, and thus, represents a novel strategy for the treatment of a variety of CNS disorders (6). In humans, microglia emerge at 4.5 weeks gestation in the meninges, ventricular rim, and plexus, and migrate from these locations to the telencephalon and mesencephalon. During this process, microglia gradually develop into a branched form, appearing in this form from as early as 12 weeks gestation (7). Microglia differentiation is regulated by the transcription factor spleen focus forming virus proviral integration oncogene (SPI1, formerly known as PU.1) and interferon regulatory factor 8 (8). Microglia self-renewal in the CNS depends on the continuous activation of colony-stimulating factor 1 receptor (CSF1R) (9, 10).

### Phenotype of microglia

Microglia produce opposing actions (i.e., neurotrophic vs. neurotoxic) depending on their activation status. Based on the classification of macrophages, microglia are usually divided into two groups: classically activated microglia (M1 type) and alternatively activated microglia (M2 type) (11). The inflammatory M1 type is usually caused by Toll-like receptors and γ-interferon signaling pathways. M1 microglia produce pro-inflammatory cytokines such as tumor necrosis factor-α (TNF-α), interleukin-6 (IL-6), IL-1, IL-1β, and chemokines, and express nicotinamide adenine dinucleotide phosphate (NADPH) oxidase and matrix metalloproteinases (MMP)-12. The anti-inflammatory M2 type plays a neuroprotective role. M2 microglia upregulate arginase 1 expression, secrete growth factors, and promote the release of anti-inflammatory cytokines such as IL-10 and transforming growth factor β (TGF-β) (12). Accumulating evidence suggests that microglia polarization is multidimensional, with extensive overlap in gene expression, rather than occurring on a simple linear spectrum (13). Single-cell RNA sequencing studies have shown that microglia can express both M1-type and M2-type activation marker genes (14). Transcriptomic studies have shown that microglia activation exhibits a broader transcriptional profile than M1 and M2 types (15). The *in vivo* phenotype of microglia is even more complex. A recent single-cell RNA sequencing study demonstrated at least nine subpopulations of microglia in the mouse brain (16). Similarly, nine microglia subpopulations have been identified in the cerebrocortex of the human brain (17). In an analysis based on the three-dimensional (3D) morphology of microglia, 10 distinct microglia subpopulations were proposed in the mouse and human brain (18). Spatial and temporal heterogeneity have also been observed at the single-cell level in mouse and human microglia (7). Moreover, distinct microglia subpopulations have been observed in specific CNS disorders, including amyotrophic lateral sclerosis (19) and Alzheimer's disease (AD) (20).

### Function of microglia

Microglia maintain CNS homeostasis in both the developing and mature brain. In the developing brain, microglia are implicated in neurogenesis, programmed cell death, synapse elimination, and neural circuit formation (21). During early development, microglia secrete neurotrophic factors to promote neurogenesis and maintain the survival and differentiation of specific neuronal lineages (22, 23). Immature neurons undergoing programmed cell death are cleared by microglia without triggering inflammatory processes (24). Microglia also remodel the CNS by selectively pruning redundant neuronal protrusions and defective synapses (25). In the adult brain, alterations in the microenvironment cause rapid and profound changes in microglia morphology and function as well as gene expression. Microglia also actively interact with neurons to influence neuronal regeneration, proliferation, and migration, and secrete neurotrophic factors such as insulin-like growth factor-1 (IGF-1) to promote the survival of neighboring neurons (26). Microglia are an important part of the innate immune sentinel network and serve as the first line of defense in the CNS. Cellular debris or dead cells can be cleared by microglia *via* phagocytosis, without triggering an inflammatory response (27). Upon invasion by pathogens, microglia respond by producing pro- and/or anti-inflammatory cytokines, chemokines, and complement proteins. These responses promote pathogen clearance but may, on occasion, elicit a strong collateral inflammatory response and exacerbate CNS pathology.

## Membrane receptors and pathways in microglia activation

### Membrane receptors

**Figure 1:**
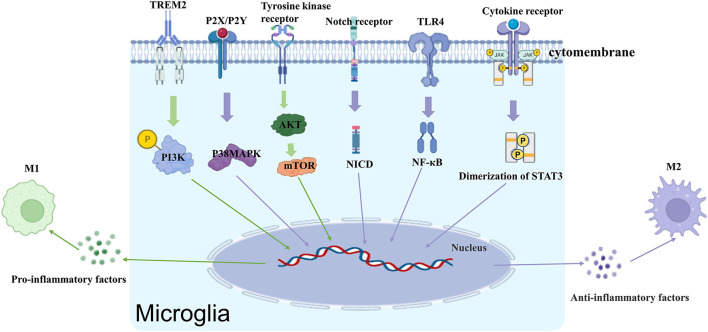
Receptors and signaling pathways that regulate microglia activation.

The activation of microglia is a complex process that involves multiple stimuli, including lipopolysaccharide (LPS), β-amyloid (Aβ), IL-1β, TNF-α, interferon (IFN)-γ, adenosine triphosphate (ATP), peptidoglycan (PG), and a variety of membrane receptors (Figure 1).

#### Toll-like receptors

TLRs play an important role in inflammation, signal transduction, and apoptosis. The TLRs that are associated with microglia activation include TLR2, 3, 4, and 9. TLR4 is the primary receptor that mediates the activation of microglia by LPS (28). Kacimi et al. found that LPS bound to TLR4 on the cell membrane of microglia to activate its downstream signaling pathways, including the nuclear factor (NF)-κB, Janus kinase-signaling and transcriptional activating protein (JAK-STAT), C-Jun amino-terminal kinase (JNK), and p38/Mitogen-activated protein kinase (MAPK) pathways (29). In a study by Capiralla et al., LPS amplified TLR4/NF-κB/STAT signaling in microglia to increase the release of TNF-α and IL-6 (30).

#### TNF receptors

Microglia express both TNFR1 and TNFR2. Exposure to IFN-γ or LPS increases TNFR1 expression in microglia (31). In the normal rat cortex, a portion of TNFR1 is located in lipid raft microdomains, Moderate TBI resulted in rapid recruitment of TNFR1, but not TNFR2 or Fas, to lipid rafts and induced alterations in the composition of signaling intermediates. Activation of TNFR1 after TBI induced the expression of caspase-8, thus initiating apoptosis and transient activation of NF-κB (32). Novel TNF-R1 inhibitors was found to significantly reduce cortical inflammatory cytokines 3 h after traumatic brain injury (TBI) and improved functional recovery following diffuse TBI in mice (33).

#### Ionotropic (P2X) and metabotropic (P2Y) purinergic receptors

Microglia express both P2Y receptors coupled to G-proteins, as well as, P2X receptors coupled to ligand gated cation channels (34). They play an important role in microglia activation, motility and paracrine signaling. P2Y12R, a microglia-specific receptor that mediates microglial chemotaxis toward site of injury (35). Activation of P2Y12R results in the opening of K+ channel and increased cAMP levels that is implicated in microglia surveillance and chemotaxis toward injury site (36). Not only that P2Y12R on microglia in the spinal cord promotes the production of pro-inflammatory cytokines through the p38/MAPK pathway, and as such, has been implicated in the maintenance of neuropathic pain (37). Research has also shown that blockade of P2X7 receptors significantly reduces nociception in animal models of chronic neuropathic and inflammatory pain (38). The P2X7R is identified as a key player in the process of microgliosis, where by driving microglial activation, it can potentially lead to a deleterious cycle of neuroinflammation and neurodegeneration (39).

#### IL-1 receptor

Microglia are the major source of IL-1 in the CNS. Upregulation of IL-1R in activated microglia plays an important role in microglia activation, neuronal damage, expression of pro-inflammatory cytokines, and degenerative CNS diseases (40). In newborn rats, LPS causes excessive microglia activation and nociceptive hypersensitivity that can persist into adulthood (41). In contrast, IL-1R antagonists can attenuate microglia activation and prevent nociceptive hypersensitivity. Silencing IL-1R can also attenuate microglia activation and reduce the expression of cyclooxyganese (COX)-2 and IL-6 (42).

#### IFN-γ receptor

Similar to astrocytes, both microglia and oligodendrocytes express IFN-γR (43). IFN-γ can produce seemingly opposing actions depending upon the specific circumstances. IFN-γ can exert protective effects by increasing phagocytosis and the clearance of neuronal debris and Aβ and inhibiting the expression of TNF-α and COX-2 (44). IFN-γ can also damage the CNS through microglia activation. In addition to activating resting microglia, stimulating the IFN-γR signaling pathway also upregulates the tyrosine kinase Lyn and purinergic P2X4 receptors of microglia, leading to abnormal neuronal excitation in dorsal horn neurons and neuropathic pain (45).

#### Peroxisome proliferators-activated receptors

PPARs are a family of ligand-activated nuclear transcription factors in the cytoplasm. PPARs are composed of three different isomers (i.e., α, β/δ, and γ) (46). PPARγ forms a heterodimer with retinol-like X receptors and binds to promoter regions to regulate their transcription (47). In a mouse study by Pan et al., the *resveratrol* malibatol A produced neuroprotective effects by activating PPARγ, which, in turn, switched microglia from the pro-inflammatory M1 type to the anti-inflammatory M2 type (48). PPARα can also interact directly with pro-inflammatory transcription factors such as NF-κB and P38MAPK to negatively regulate their targets and produce anti-inflammatory effects through a non-DNA-dependent mechanism (49).

#### Others

β-Adrenergic receptor antagonists have been shown to block CD11b upregulation, increase the production of a variety of pro-inflammatory cytokines by microglia, and attenuate anxiety-like behavior in mice subjected to repeated social defeat (50). Studies have also found that CXCL10, a chemokine released by neurons after brain injury, activates microglia to promote the dissipation of innervated dendrites from the injury site; these effects were obliterated in CXCR3 knockout mice. Other chemokines that have been implicated in microglia activation include C-C chemokine receptor 2 (CCR2), CXC Chemokine Receptor 3 (CXCR3), and chemokine (C-X3-C motif) receptor 1 (CX3CR1) (51).

### Signaling pathways

#### Notch signaling pathway

The Notch pathway is highly conserved in a variety of organisms and is involved in the development of almost all organ systems. Upon binding with ligands, activated Notch receptors on the cell surface of microglia increase protein hydrolase cleavage and the release of intracellular segments of Notch into the nucleus, where they bind to the transcriptional repressor RBP-Jκ to activate the transcription of target genes and regulate cell proliferation, differentiation, and apoptosis (52). The Notch signaling pathway has been shown to mediate the release of TNF-α, IL-1β, IL-6, and other pro-inflammatory factors in activated microglia, promoting cell proliferation and migration. The Notch signaling pathway also promotes the activation of various key pathways such as NF-κB, p38 MAPK, and JNK in microglia by regulating the expression of the deubiquitinating enzyme CYLD, thereby facilitating microglial activation (53).

#### MAPK, ERK, and NF-κB pathways

The MAPK, ERK, and NF-κB pathways have been shown to play key roles in the transcriptional and post-transcriptional regulation of iNOS and TNF-α gene expression in LPS-activated microglia (54). MAPK is an important molecule that carries signals from the cell surface to the nucleus. Previous studies have shown that the activation of p38MAPK signal pathway is closely related to neuroinflammation caused by activation of microglia in ischemic stroke (55). At the molecular level, MicroRNA22, Apelin-12 can inhibit p38MAPK signaling pathway to inhibit the microglia activation to improve ischemic brain injury (56, 57). After rat primary microglia were treated with thrombin, microglia were activated, iNOS expression increased, and ERK phosphorylation was induced (58). After treatment of lipopolysaccharide-activated BV-2 microglial cells with ERK inhibitor SCH772984, the release of nitric oxide was inhibited, and the phosphorylated ERK decreased (59). The role of NF-κB, a key factor in transcriptional regulation, is crucial in the regulation of inflammatory response and immune stress (60). It has been shown that microglia activation is closely related to NF-κB signaling pathway (61). Stepharine improves the outcome of middle cerebral artery ischemia rats by inhibiting TLR4/NF-κB signaling pathway, attenuating neuronal damage and suppressing microglia hyperactivation (62). Quercetin reduces microglia activation by inhibiting NF-κB signaling pathway and plays a neuroprotective role against brain injury caused by ischemia and hypoxia (63).

#### Src family kinases

Src family kinases (SFKs), a class of non-receptor tyrosine kinases, play an important role in regulating cell proliferation, differentiation, metabolism, the immune response, and signal transduction. Evidence indicates that epidemic B encephalitis virus (JEV)-stimulated Src activation promotes TNF-α and IL-1β expression by initiating Src/Ras/Raf/ERK/NF-κB and Src/Raf/ERK/NF-κB in microglia (64).

#### PI3K/AKT signaling pathway

Abundant research shows that a central role for the PI3K-AKT signaling pathway in health and disease (65, 66). In addition, activated AKT is vital for initiating immune responses, as evidenced by the functional defects observed in various leukocyte subsets when PI3K and AKT are ablated in experimental rodent models (67). For example, *in vivo* systemic challenge with LPS in rodents leads to an increase in phosphorylation of AKT *via* PI3K signaling in the CNS, which induces activation of microglial populations in the cortex, hippocampus, and thalamus, and is associated with exacerbated release of the pro-inflammatory factors TNF-α, IL-1β, iNOS, and IL-6 (68, 69).

#### Other pathways

Apelin-13 inhibits LPS-induced activation of M1-type microglia by downregulating the STAT3 signaling pathway (70). Binding of LPS to TLR4 receptors on the microglial membrane surface recruits downstream signaling molecules by activating the MyD88/IRAK/TRAF6 signaling cascade, which, in turn, causes IKKα/β phosphorylation, IκB-α phosphorylation, IκB-α degradation, NF-κB nuclear translocation, NF-κB p65 phosphorylation, and other signaling cascades, ultimately enhancing NF-κB transcriptional activity (71–73). Mammalian target of rapamycin (mTOR) is a member of a conserved serine/threonine kinase family that is implicated in many fundamental biological processes, including gene transcription, protein translation, and ribosome synthesis (74). mTOR interacts with the IKK complex and increases IKK activity, which, in turn, promotes NF-κB transcriptional activity (75, 76). Endogenous α-KG inhibits microglia activation by suppressing the mTOR/NF-κB pathway. TNF-α-sensitive pericytes release IL-6 to activate microglia through the synergistic action of the IκB-NF-κB and JAK-STAT3 pathways (77).

## Microglia activation and CNS disorders

### Brain injury

#### Ischemic stroke

Ischemic stroke, also known as cerebral infarction, is the sudden interruption of cerebral blood flow in local brain tissue due to various reasons; it accounts for 85% of all strokes. Ischemic stroke leads to necrosis of local brain tissue and corresponding neurological deficits. Microglial activation has traditionally been considered to be detrimental in ischemic stroke (Table 1), and thus, has been extensively studied. However, a growing body of evidence suggests another side to the coin: microglial activation may be critical for neurogenesis, angiogenesis, and synaptic remodeling, thereby facilitating functional recovery after cerebral ischemia. When ischemic stroke occurs, the lack of blood sugar and oxygen in brain tissue leads to the death of neurons, the release of reactive oxygen species, the activation of complement, the up-regulation of endothelial cell adhesion factors, and the dead cells release danger signals to the brain microenvironment, including HMGB1 and ATP, to activate microglia (96). Overall, microglia activation consists of two phases: the M2-like anti-inflammatory phenotype, which is predominant from day 0.5 to 7 after cerebral ischemia, and the M1-like pro-inflammatory phenotype, which is predominant from day 7 to 14. M1-type microglia produce pro-inflammatory mediators, including TNF-α, IL-1β, interferon-c (IFN-c), IL-6, iNOS, MMP-9, and MMP-3 (78). On the one hand, these pro-inflammatory factors and cytotoxic substances secreted by M1-type microglia may directly or indirectly damage neuronal survival, prevent neurogenesis, and aggravate long-term neurological deficits after brain injury (97). On the other hand, they promote the formation of inflammatory microenvironment, which is conducive to the transformation of unpolarized microglia into M1 type, forming pro-inflammatory feedback and promoting the pathological process of cerebral ischemia, which is also unfavorable for neurons after ischemic stroke. In addition, M1-type microglia can prevent axon regeneration (98) and exacerbate oligodendrocyte death. This is not conducive to central nervous system recovery. In contrast, M2-type (alternative) microglia are characterized by the production of pro-angiogenic and anti-inflammatory molecules, including IL-10, TGF-β, insulin-like growth factor, and vascular endothelial growth factor (VEGF) (78). Anti-inflammatory factors secreted by M2-type microglia cells can reduce inflammation and improve ischemic brain injury. The neurotrophic factors secreted by it can promote the proliferation of neural precursor cells, accelerate the migration of neuroblasts (99), promote the maturation of new neurons, and promote the repair of the central nervous system. In addition, after ischemic stroke, M2-type microglia and macrophages, compared with M1 phenotypes, have a stronger phagocytosis capacity for necrotic neurons to remove damaged neurons, prevent secondary inflammatory reactions, and provide survival space for new neurons to maintain the homeostasis of the central nervous system under pathological conditions.

**Table 1:** Involvement of microglia in CNS disorders.

**Disease**	**Microglia participation in the process of the disease**	**Activation pathway**
Ischemic stroke	M2-like anti-inflammatory phenotype was predominant at 0.5–7 days after cerebral ischemia, and M1-like pro-inflammatory phenotype was predominant at 7-14 days after cerebral ischemia (78).	Notch; TLR/NF-κB; mTOR/STAT3 (79–81)
Hemorrhagic stroke	Microglia were activated in the acute phase and maintained for more than 21 days (82).	Acute phase: TLR4/MyD88 (83) Late-onset phase: RLR4/TRIF (84)
TBI	Microglia were activated in the acute phase and the chronic activation state was maintained for decades (85).	TLR/NF-κB; (86) PPARs (87)
ALS	The hyperplastic infiltration of microglia occurred in the early stage of the disease and was accompanied by the final stage of ALS (88).	CD14; TLR/NF-κB (89)
AD	Microglia are activated and induce abnormal Tau phosphorylation, which accelerates the progression of AD (90).	TLR/NF-κB; (91). TREM2/DAP12 (92).
Neuropathic pain	Activated microglia maintain and exacerbate neuropathic pain by aggravating the inflammatory response (93).	P2X4/P38MAPK; (94). α7n ACh R/BDNF/Trk B (95).

#### Hemorrhagic stroke

Hemorrhagic stroke is characterized by a sudden-onset cerebral aneurysm rupture or vascular leakage, leading to cerebral blood circulation disorders. Patients with cerebrovascular diseases may suffer from cerebral arterial stenosis, occlusion, or rupture due to various causes, thus resulting in acute cerebrovascular hemorrhage. Patients exhibit symptoms and signs reflective of temporary or permanent cerebral dysfunction. Hemorrhagic stroke accounts for 15% of all strokes. After intracerebral hemorrhage, a large amount of blood enters the brain tissue to form hematoma, resulting in space occupying lesions and release cell components, thrombin, chemokines and other bioactive substances, activating microglia cells through different ways. M1 microglia are largely pro-inflammatory and release high levels of pro-inflammatory factors upon activation, including IFN-γ, TNF-α, and IL-1β. This excessive inflammatory response is an important source of secondary injury in hemorrhagic stroke. M1 microglia also secrete large amounts of reactive oxygen species (ROS) and protein hydrolases, including heme oxygenase-1 (HO-1), MMP, and iNOS. ROS accumulation increases the expression of apoptotic genes and inflammatory mediators, which, in turn, induces apoptosis and disrupts the blood-brain barrier (BBB) to promote secondary injury (100). Iron accumulation caused by HO-1 overexpression also aggravates secondary injury by increasing lipid peroxidation and free radical formation. Overproduction of NO due to iNOS activation increases peroxynitrite to disrupt the BBB (101). Overexpression of MMP-2 and MMP-9 can also disrupt the BBB by injuring the tight junctions between capillary endothelial cells and the basement membrane (102). Activated M2-type microglia secrete anti-inflammatory factor IL-10 and up-regulate its phenotype markers to play an anti-inflammatory role (103), and can produce specific trophic factors to promote the proliferation of neuroprecursor cells and the migration of neuroblasts, and facilitate the functional integration of new neurons into the existing neural circuits to assist the recovery of nerve function after injury. Moreover, increased secretion of protective molecules such as IL-10 can promote axon regeneration and recovery (104).

#### TBI

M1-type and M2-type microglia exhibit different temporal patterns in a controlled cortical injury (CCI) model (105). Microglia can remain chronically activated for years after the acute inflammatory process has subsided. In patients with TBI, microglia activation was found to persist for decades (85, 106). The activation pattern of microglia also varies spatially. Microglia, macrophages, and astrocytes are predominantly located around the lesion site in the acute phase but spread to distant brain regions by 1-year post-injury (107). These findings highlight the long-lasting impact of TBI and suggest the presence of persistent neuroinflammation at the site of the injury and beyond. Studies of the heterogeneity of brain inflammation after injury, especially those focusing on the microglia polarization phenotype, are needed to develop treatments based on the regulation of microglia polarization.

### Neurodegenerative diseases

#### AD

**Figure 2:**
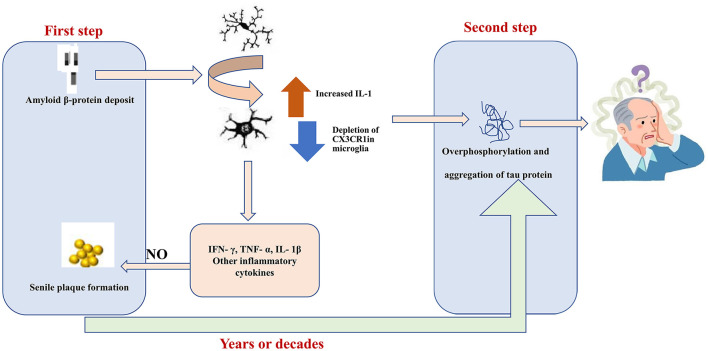
Aβ1-42 activates MG through CD36 and the toll-like receptor TLR2-TLR6 heterodimer. Subsequently, the expression levels of pro-inflammatory factors, interleukin (IL)-1β, and tumor necrosis factor (TNF)-α were significantly increased. In which nitric oxide produced by activated MG can lead to nitration of Aβ peptide 10Y, and this modification promotes the formation of senile plaques. Elevated IL-1 levels in MG caused by depletion of CX3C chemokine receptor 1 (CX3CR1) lead to hyperphosphorylation of tau. Hyperphosphorylated tau aggregates are formed in the nerves and eventually lead to neuronal cell death. The gap between these two waves of destruction could be years or even decades.

The characteristic pathological features of AD include senile plaques, neurogenic fiber tangles, neuroinflammation, and neuronal loss (Figure 2). Deposition of Aβ secreted from neurons and the formation of extracellular senile plaques mark the first wave of damage to neurons. This is followed by the formation of neurogenic fiber tangles (hyperphosphorylated Tau aggregates) within nerves, which eventually leads to neuronal death. The interval between the two waves of damage may be years or even decades. Aβ has been found to induce the aggregation of microglia and infiltration around amyloid plaques. *In vitro* experiments have demonstrated that Aβ1-42 activates microglia *via* CD36 and TLR2-TLR6 heterodimers (108), followed by excessive expression of the proinflammatory factors IL-1β, TNF-α, molecularly imprinted polymer-1α, and monocyte chemotactic protein-1 (109). Aβ can also directly interact in a concentration-dependent manner with the amyloid precursor protein (APP), resulting in the activation of microglia and secretion of the inflammatory factor TNF-α (110). In addition, NO production by activated microglia can lead to nitration of Aβ peptide 10Y, a modification that promotes the formation of age spots (111). Activated microglia produce pro-inflammatory cytokines (e.g., TNF-α) and increase Aβ deposition by upregulating β-secretase, a rate-limiting enzyme for Aβ production (112). In another study, activated microglia and damaged neurons were found to release high mobility group protein B1 (HMBG1), which, in turn, was found to bind to Aβ to form Aβ oligomers, inhibit the clearance of Aβ by microglia, and increase the release of pro-inflammatory factors and oxygen radicals to aggravate neuroinflammation (113). Furthermore, Tau hyperphosphorylation and aggregation are driven by microglial activation (114, 115). Specifically, elevated IL-1 caused by CX3CR1 depletion in microglia leads to increased Tau hyperphosphorylation (116). Together, these studies strongly suggest that microglia activation may be an important link between the two waves of neuronal cell injury, i.e., Aβ deposition and hyperphosphorylated Tau protein aggregation, in AD.

#### Parkinson's disease

The main features of Parkinson's disease (PD) pathologies are selective degeneration and loss of dopamine (DA) neurons in the dense substantia nigra, deposition of α-synuclein (α-Syn), and the presence of Lewy bodies (LB) (117). Currently, there is no effective treatment for PD. Microglia-mediated neuroinflammation plays an important role in the pathogenesis of PD, controlling the survival and death of DA neurons. Activated MG was found in the midbrain substantia nigra dense zone (SNpc) of PD patients by immunofluorescence co-localization, and microglia-produced inflammatory factors cause the loss of DA neurons in the midbrain substantia nigra (118). When microglia are aberrantly activated, it produces inflammation and engulfs connections between brain cells, inducing neuronal death and resulting in brain damage triggering PD. Xia et al. found that microglia exosomes are involved in the α-Sny aggregation in the pathological process (119). Activated microglia releases exosomes that promote the infection of insoluble α-Sny with normal cells and propagate the aggregation of pathological α-Sny, which is accelerated by pro-inflammatory factors released in response to microglia inflammation, and the use of GW4869 (an inhibitor of sphingosine phosphodiesterase) significantly reduces the number of exosomes and decreases α -Sny proliferation and aggregation, leading to the possibility of targeting microglia exosomes for PD treatment (120). Also, age-related changes in the inflammatory response, as well as genetic and environmental factors, may contribute to the prevalence of pro-inflammatory microglia and affect the susceptibility to microglia activation, which may further influence the pathogenesis and lead to progressive neuronal loss.

#### Amyotrophic lateral sclerosis

Gerbino et al. (121) found large numbers of activated microglia and astrocytes in the brainstems and spinal cords of transgenic SOD1 ALS model mice as well as in human ALS patients. Positron emission tomography (PET) imaging also revealed activated microglia in the motor cortex, thalamus, and pons of ALS patients. Activated microglia produce large quantities of pro-inflammatory factors, such as TNF-α, monocyte chemotactic protein-1 (MCP-1), and MMP-9, as well as glutamate. This, in turn, activates glutamate receptors to produce excitotoxic damage to motor neurons. Activated microglia also release large amounts of chemokines and pro-inflammatory factors to activate surrounding astrocytes. The reciprocal interaction between activated microglia and reactive astrocytes forms a positive feedback loop to aggravate neuronal damage (122). Microglia can communicate with other cells by releasing microRNAs (miRNAs) which play a role in the post-transcriptional regulation of gene expression (123). Dysregulation of miRNAs has been observed in ALS patients, accompanied by inflammation and an altered microglial cell phenotype. Christoforidou et al. (124) found that microglia are the most likely source of miRNA dysregulation in ALS patients and correction of miRNA dysregulation through modulation of microglia function may be a new strategy for the treatment of ALS.

#### Multiple sclerosis

Multiple sclerosis is an inflammatory demyelinating disease mainly involving the CNS white matter of the central nervous system, and its pathological features include CNS inflammation, gliosis, demyelination and neurodegeneration (125). The experimental autoimmune encephalomyelitis (EAE) is the ideal animal model for the study of MS, which also exhibited similar pathological characteristics as MS. In EAE mouse model microglia release proteases, pro-inflammatory cytokines, ROS and reactive nitrogen species (RNS), and recruit reactive T lymphocytes, thus producing toxic effects on neurons and oligodendrocyte precursors. Targeted knockout of TGF-β-activated kinase 1 alleviates central nervous system inflammation and axonal and myelin damage (126). Other studies have shown that microglia cells are closely related to active demyelinating lesions (127). In fact, microglial activation can be detected before MS lesions damage the myelin sheath. Both white matter demyelination and diffuse axon injury have been shown to be associated with microglial activation. In addition, activation of microglia can be detected in the white matter prior to leukocyte infiltration, blood-brain barrier disruption, or demyelination (128). Activated microglia cause myelin loss and axonal damage. In addition, microglia may also affect gray matter. In MS patients, significant microglia activation is consistent with cortical damage in patients (129).

### Neuropathic pain

Upon neuropathic pain, nociceptive neurons are excited and release a variety of neurotransmitters (e.g., glutamate, ATP, and substance P) to increase the release of various cellular inflammatory factors such as MMPs, MCP-1/CCL-2, CX3CLI, and Toll receptor agonists (123). These pro-inflammatory factors activate microglia, which, in turn, release a variety of neuroexcitatory and pain-sensitizing substances. The expression of P2X4 receptors on the surface of microglia is regulated at the post-translational level by the CC chemokine receptor chemotactic cytokine receptor 2 (130). In addition, spinal microglia respond to extracellular stimuli through intracellular signaling pathways such as the mitogen-activated protein kinase, p38, and extracellular signal-regulated protein kinases pathways. After long-term repeated action of injurious stimulus signals on microglia, microglia cells will continue to be activated, increasing the degree of M1 polarization, and then evolves into chronic pain. This is one of the reasons why chronic neuralgia is so difficult to cure. Neuroinflammation mediated by microglia M1 polarization and its subsequent production of pro-inflammatory factors plays an important role in mediating neuropathic pain. At the same time, with the regression of inflammation in the later stage of this model, activated microglia began to transform from M1 type to M2 type, promoting the production of anti-inflammatory factors such as IL-4 and IL-10, helping to restore normal nerve function and transform the pain state. Thus, the polarization state of microglia cells is associated with pain. The status can appear dynamic changes in the occurrence and development of chronic neuropathic pain. Reasonable regulation of microglia polarization in the occurrence and development of chronic neuropathic pain is expected to be a potential strategy for the treatment of chronic neuropathic pain.

## Therapeutic implications

### Modulation of the inflammatory response

#### Non-steroidal anti-inflammatory drugs

Molecular targets of classical NSAIDs (i.e., aspirin, ibuprofen, naproxen, diclofenac, and sulforaphane) include COX-1 and COX-2. COX-1 is constitutively expressed in most tissues, including microglia, whereas COX-2 is inducible by inflammatory processes. The selective COX-2 inhibitor celecoxib attenuates LPS-induced systemic inflammation and brain white matter injury in neonatal rats (Table 2); this action is associated with a reduction in the number of activated microglia and astrocytes, a decrease in the IL-1β and TNF-α secretion levels, and inhibition of phosphorylated p38MAPK (139). In the Tg2576 transgenic AD mouse model, long-term administration of ibuprofen attenuates the activation of microglia, the release of IL-1β, axonal dystrophy, and amyloid plaques (140). Although observational epidemiological data show that NSAIDs have protective effects, and there is evidence that anti-inflammatory therapy has biological paradoxical effects, the first large primary prevention study of naproxen and celecoxib [Alzheimer's Disease Anti-inflammatory Prevention Test (ADAPT)] does not support the hypothesis that celecoxib or naproxen prevent adult AD with family history of dementia (141). A 2-year primary prevention trial [effect of naproxen treatment on pre-symptomatic Alzheimer's disease (INTREPAD)] showed that naproxen had no benefit in slowing the progression of pre-symptomatic AD compared with placebo (142). In addition to the gastrointestinal and cardiovascular safety issues most often mentioned by COX-2 selective inhibitors, other drug side effects caused by its inhibition of COX-2 but not COX-1 have also gradually attracted clinical attention. This reduces the therapeutic advantage of COX-2 selective inhibitors.

**Table 2:** Therapeutic strategy targeting microglia for CNS disorders.

**Molecular or cellular target**	**Pharmacological intervention**	**Results**	**Reference**
**(i) Inflammatory response**			
Immuno modulator	Minocycline	Anti-apoptotic, anti-inflammatory, and antioxidant effects on several PD models.	(131)
COX2	NSAIDs	Prevention of inflammation and dyskinesia in the rotenone rat model.	(132)
**(ii) Phenotypic transformation**			
Induces an M1 to M2 switch in microglia phenotype	Progesterone	Progesterone treatment reduced neurobehavioral deficits in the mouse demyelinating model.	(133)
Promoted transformation of microglial M1 phenotype to M2 phenotype	Fractalkine	FKN partially recovered the spatial memory of irradiated mice.	(134)
**(iii) Microglia depletion and regeneration**			
Microglia depletion strategy	CSF1R inhibitors	Attenuates neurological abnormalities and brain edema.	(135)
Microglia repopulation strategy	Multipotent adult progenitor cells	Improved the BBB after traumatic brain injury, weakened the activated microglia macrophages in the dentate gyrus, and improved cognitive behavior.	(136)
**(iv) Receptors and pathways**			
NF-κB, MAPK	Metformin	Ameliorated neurological deficit, cerebral edema, and neuronal apoptosis in rats following TBI.	(137)
Akt/mTOR/ STAT3 signaling pathway	6-gingerol	Reduced the size of infarction and improved neurological functions in the ischemia brain damage rat model.	(138)

#### Minocycline

Minocycline and related tetracyclines possess anti-inflammatory activity and neuroprotective effects in several models of CNS disorders. In a transgenic McGill-Thy1-APP mice for Alzheimer's disease, early treatment with minocycline was found to reduce iNOS and COX-2 expression (143), protect hippocampal neurogenesis (144), and improve cognition (145). However, inhibition of microglial activation failed to improve cognition if minocycline treatment was initiated after the onset of Aβ deposition (146). The neuroprotective effect of minocycline on animals has initiated the study of its clinical efficacy in AD and PD patients, but the results are uncertain. In addition, the safety data of long-term use of doxycycline and minocycline in these patients are insufficient. Safety issues should be considered from two levels. The fact that AD/PD patients (especially the changes of intestinal flora caused by antibiotics and their consequences), as well as AD and PD are universal incurable diseases that require long-term antibiotic treatment every day, constitutes a worldwide threat of bacterial resistance to these antibiotics (147).

#### Natural compounds

Plant-derived natural compounds are a major source of novel neuroprotective agents. Such compounds include flavonoids, glycosides, phenolics, terpenoids, quinones, alkaloids, lignans, coumarins, chalcone, stilbene, and others (biphenyl, phenylpropanoid, oxy carotenoid). These compounds can reduce the expression of neurotoxic mediators (NO, PGE2, iNOS, and COX-2) and pro-inflammatory cytokines (IL-6, TNF-α, and IL-1β), down-regulate inflammatory markers, and prevent neural damage (148). Hyperoside, a natural flavonoid glycoside, has anti-neuroinflammatory properties. In MPTP-induced PD mice, hyperoside inhibited the activation of glia and reduced the secretion of inflammatory factors, protecting DA neurons and reversing the motor dysfunction caused by MPTP (149). Morin is a natural flavonol extracted from plants from the Moraceae family. It can reduce the expression of TNF-α, IL-1β, IL-6, and increased the expression of brain-derived neurotrophic factor in the hippocampus of Aβ1-42-injected rats, attenuated memory deficits in a rat model of Alzheimer's disease (150). Although various preclinical studies have been conducted on the role of natural compounds in neurodegenerative diseases, little is known about their exact effects in humans, and further clinical trials are needed to confirm the neuroprotective efficacy of this natural compound and to assess its safety (151). At the same time, natural compounds have some limitations, such as their pharmacokinetic properties and stability, which hinder their clinical development and use as drugs.

### Phenotype transformation

In many studies, anti-inflammatory drugs (e.g., aspirin, celecoxib, and naproxen) have failed to prevent or treat neurodegenerative diseases (152–154), suggesting that inhibition of M1 microglia alone is not sufficient; concomitant M2 microglia activation may be needed. In a mouse model of demyelination induced by a neurotoxic copper reagent (CPZ), progesterone reduced the level of demyelination and had an anti-inflammatory role in CNS demyelination by inducing M2 microglia polarization and suppressing the M1 phenotype through the inhibition of NF-κB and NLRP3 inflammasome (155). The latest studies in animal stroke models demonstrate that curcumin can exerts their anti-inflammatory effects *via* attenuation of brain proinflammatory microglia M1 polarization while promoting anti-inflammatory microglial M2 polarization. As a result, stroked animals treated with curcumin have significantly fewer brain active M1 microglia, smaller brain infarct volume, better functional recovery, and better survival rate (156). Semaglutide, a long-acting glucagon-like peptide-1 (GLP-1) receptor agonist, was found to inhibit the neuroinflammatory response and promote neural repair by promoting microglia conversion to the M2-like phenotype and inhibiting M1-like phenotype conversion (possibly mediated by the NF-κB pathway p65) in rats with cerebral ischemia-reperfusion (157). Although these targeted microglia therapies can improve the disease symptoms to a certain extent, they do not achieve precise treatment effects due to the heterogeneity of microglia. Thus, techniques and methods to target functionally-specific microglia to achieve precise disease treatment must be explored in the future.

### Microglia depletion and regeneration modulation

Accumulating evidence suggests that microglia depletion may represent a therapeutic approach for the prevention and treatment of CNS disorders that involve neuroinflammation. The CSF1R inhibitor PLX3397 can deplete microglia and attenuate brain injury and neurological deficits in an animal model of hemorrhagic stroke (158). Interestingly, microglia regeneration has been shown to promote hippocampal neural regeneration and improve spatial learning through the IL-6 signaling pathway in the early stages of TBI (69). In the *CaM/Tet-DTA* mouse model of induced neuronal loss, microglia depletion and then regeneration promotes functional recovery (159). Thus, in preclinical studies, depletion of microglia by removing the trophic factors required to maintain them, and subsequent repopulation with a more beneficial microglia phenotype, may produce therapeutic effects. The key problem with this approach is the unknown risk of microglial depletion and the ethical concerns of repopulating microglia in the CNS. In addition, it has been found that depletion and regeneration of microglia can induce the increase of gray matter microglia, neuronal death in somatosensory cortex and atax-like behavior.

### Microglia activation-related receptors and signaling pathways intervention

Activation of microglia and astrocytes exacerbates glyoxylate deprivation in rats with ischemia-reperfusion injury to the brain; P2X7 receptor antagonists can attenuate memory deficits and inflammatory factor production in the hippocampus and improve animal survival (160). *TLR4* gene mutation blocks the activation and release of pro-inflammatory factors by microglia (161). Metformin inhibits the translocation of NF-κB p65 from the cytoplasm into the nucleus and the phosphorylation of ERK1/2 and p38 MAPK inhibits microglia activation and reduces the production of pro-inflammatory cytokines to improve neurobehavioral function in an animal model of TBI (137). The 20-hydroxyeicosatetraenoic acid (20-HETE) inhibitor HET0016 inhibits microglia activation and reduces the release of pro-inflammatory factors through the NF-?B signaling pathway, demonstrating promising protective effects against TBI in the developing brain (162). It is generally believed that the anti-inflammatory effect of α7 nAChR is mediated by NF-κB and JAK2/STAT3 (163). Nicotine can significantly decrease the levels of TNF-α and IL-1β in the hippocampal CA1 region of ischemic stroke rats by up-regulating α7 nAChR and inhibiting microglia inflammation (164). The signal pathways and molecular mechanisms involved in the activation and polarization of microglia are complex and diverse. The activation of microglia can be regulated by targeting different pathways and molecules. However, the exact mechanism of microglial activation pathway, especially the molecular basis of signal transduction in the process of activation, is not completely clear. At the same time, different signal pathways are also linked and can be regulated. These results in that the treatment strategy of treating related diseases by interfering with microglia activated receptors and signal pathways cannot be fully effective.

### Challenges

Despite the accumulating preclinical evidence suggesting that the targeting of microglia could potentially be applied in the treatment of CNS disorders, numerous obstacles remain before this approach can be translated into clinical use. First, the phenotypic switch of microglia exhibits complex and varying spatiotemporal patterns during the course of CNS disorders. Second, the mechanisms underlying the regulation of microglia activation and phenotypic switch are implicated in many other biological processes and functions. Third, the tools used in preclinical studies to selectively manipulate microglia activation are often based on genetic approaches (e.g., knockout and small interfering RNAs), and thus, lack specificity. Fourth, the impacts of age and sex differences on microglia function have been largely overlooked in preclinical studies. Fifth, preclinical animal models often do not have solid construct validity and do not capture the heterogeneity of human disease. Finally, alteration of the microglia phenotype, loss of neuroprotective function, and acquisition of neurotoxic function are complex and may vary with the stage and severity of CNS disorders.

## Conclusion and future perspectives

In recent years, there has been increasing interest in studying the activation of microglia cells because of their potential role in the development of neurodegenerative diseases and related impairment disorders in these patients. This paper reviews the role of microglia in neurological diseases and injuries. As the main innate immune cells of the central nervous system, microglia cells are important immune cells in the CNS, playing a variety of functions such as proinflammatory, anti-inflammatory, chemotactic, phagocytotic and neuroprotective in nervous system diseases and injuries.

At present, With the development of neuroimaging, three-dimensional modeling, genomics, transcriptomics, proteomics and other technologies, combined with the study of temporal dynamics, spatial microenvironments and complex signal networks, we will have a more comprehensive and in-depth understanding of the role of microglia. This provides insight into the different distribution of microglia activation phenotypes at the injury site and the time course by which inflammation spreads to other areas of the brain, such as the thalamus.

The dual role of microglia cells often brings negative effects and is not conducive to the repair of the nervous system after injury. Recent studies have shown that simple inhibition of microglia activation has only a limited beneficial effect, and inhibition of M1-like responses can be harmful (165). Furthermore, as seen in clinical trials of AD, pharmacological treatments of anti-inflammatory agents tested in several models may not have similar effects when administered in patients with TBI, thus limiting the potential therapeutic effect (166). Therefore, it will become the focus of future research to continue to explore the rules and mechanisms of microglia activation and explore new ways to control the changes in microglia cell function. At the same time, the current studies are based on animal models, and it is unclear whether the characteristics of rodent cells apply to humans. Moreover, the controversy about the classification of microglia cells is fierce at the present stage, so strengthening the understanding of microglia cells and rational typing of them will also become the focus and hot spot of future research. Therefore, future studies must consider microglial cell polarization as a therapeutic strategy, evaluating several markers in target cells within a sufficient time window to show long-term positive results.

## Author contributions

JQ wrote the main manuscript text. XC and ZM prepared table and figures. SS did language modification. All authors reviewed the manuscript.
